# Dog owners’ intention to control rabies and their willingness to pay for rabies vaccine in Northwestern Ethiopia

**DOI:** 10.1371/journal.pgph.0003974

**Published:** 2025-03-11

**Authors:** Fentahun Mengie, Wudu Temesgen Jemberu, Yimer Mulugeta, Wassie Molla, Sefinew Alemu Mekonnen

**Affiliations:** 1 Quara district livestock development office, Gondar, Ethiopia; 2 Department of Veterinary Epidemiology and Public Health, College of Veterinary Medicine and Animal Sciences, University of Gondar, Gondar, Ethiopia; 3 International Livestock Research Institute, Addis Ababa, Ethiopia; 4 Ethiopian Public Health Institute (EPHI), Addis Ababa, Ethiopia; LSE: The London School of Economics and Political Science, UNITED KINGDOM OF GREAT BRITAIN AND NORTHERN IRELAND

## Abstract

Rabies is a viral disease that affects both animals and humans. Effectiveness of a rabies control measures depends on collaboration of dog owners. This study was conducted in North West Ethiopia to understand the intention and willingness to pay (WTP) of dog owners related to rabies control and factors affecting rabies control measures. Data was collected from 423 randomly selected dog owners in four Gondar zones, North West Ethiopia. The theory of planned behavior was used to explore the intention of dog owners towards implementing rabies control measures. Contingent evaluation method was used to evaluate WTP for rabies vaccine. Logistic regression analysis was used to test association with factors. Large majority of dog owners had positive intention to control rabies by vaccination of dogs (97% free of charge, 93% with fair price) and 94% by leashing. Majority of dog owners had a positive attitude (AT), positive subjective norms (SN) and positive perceived behavioral control (PBC) towards controlling rabies. Dog owners’ AT and SN were positively associated with intention to vaccinate dogs. Male dog owners and dog owners belonging to lower age groups had significantly higher AT towards leashing dogs. Sixty two percent of dog owners were WTP for the suggested price bid amounts; but, in general, their number decreased when price of the vaccine increased. The mean WTP for rabies vaccine was 59.25 Ethiopian Birr (1.14 USD) per year. Monthly income was associated (P < 0.05) with WTP for rabies vaccine. Dog owners who had higher income had higher odds but those who did not vaccinate their dogs in the last two years had lower odds of WTP for rabies vaccine. Intervention directed at changing AT and SN is important to increase intention of dog owners; nevertheless subsidizing rabies vaccine increase dog owners participation in dogs vaccination campaign.

## Introduction

Rabies is a disease of warm blooded animals caused by a negative-stranded RNA virus of the genus *Lyssavirus* in the family *Rhabdoviridae* [1]. Rabies virus is among twelve distinct lyssaviruses and is the most important one in affecting public and animal health [2]. Rabies is a global zoonotic disease that lacks satisfactory treatment and kills 50,000–70,000 people annually [3]. The main transmission route of rabies virus to humans is through animal bites, especially of dog bite [4]. In developing countries, majority of human rabies is through bite of unvaccinated dogs and cats or bite of infected wildlife [5].

Many steps have been taken to control and elimination of dog-transmitted human rabies. At the global level, FAO, OIE and WHO declared rabies a priority disease. In December 2015, the global rabies meeting in Geneva, Switzerland, developed a framework for the elimination of dog-mediated human rabies. The strategic vision of this framework is to reach zero human deaths from dog rabies by 2030 [6]. The most effective strategy availale to prevent rabies in humans and livestock is preventive vaccination of dogs [3,7]. Immunization of dogs and achieving population immunity levels sufficient to inhibit rabies transmission remains to be the main feasible method [8]. Dog vaccination coverage in Ethiopia is far lower than the standard WHO recommendation of 70% vaccination coverage in urban areas [9] and even was nonexistent in rural communities [10]. One reason is costs of vaccination; only owners who can afford the vaccination costs vaccinate their dogs. In the last decade, there is increasing interest of the Government to control rabies [11,12]. Currently, prevention, control and elimination of rabies are being implemented to achieve the national and global ‘zero human deaths from dog rabies by 2030’. Among the several initiatives, annual mass dog vaccination campaign is being implemented. From 2022 to 2023, more than 357,617 dogs were vaccinated. In two campaigns, from March-April 2024, 7780 owned dogs were vaccinated. However, the success of rabies elimination through vaccination of dogs depends on the commitment and collaboration of the stakeholders’ involved [13] and also with the willingness, commitment and collaboration of dog owners to change their behavior [14].

Ethiopia has an estimated more than 5 million dogs and 150,000 cats [15]. Many households own dogs usually for guarding property; it is estimated that there is one owned dog per five households [16]. There are also a lot of free-roaming (stray dogs) in which their number is not known. Ali outlined that the density of stray dogs is high in urban areas, 55 dogs per football pitch while it was 6 dogs per football pitch in rural areas [17]. From the dog population in Addis Ababa that was estimated to be 230,000 - 300,000, 30% were owned while 70% were stray dogs [18]. This high population of dogs with poor management contributes for high prevalence of canine rabies. Thousands of people are infected with rabies every year. Although the actual number of deaths caused by rabies is unknown because of under reporting [19], an estimated 2,700 people die per year, which is one of the highest rabies death rates in the world. Rabies profile in Ethiopia had shown 12 exposure cases per 100,000 population and 1.6 rabies deaths per 100,000 populations [20]. In a study in North Gondar Zone of Ethiopia, we found an annual estimated rabies incidence of 2.33 cases per 100,000 populations [21].

Rabies is a problem in domestic animals. Jemberu et al. reported 412.83 cases per 100,000 in dogs, 19.89 cases per 100,000 in cattle, 67.68 cases per 100,000 in equines, and 14.45 cases per 100,000 in goats [21]. In his survey, Mamo reported rabies cases of 67%, 11% and 10%, in dogs, cats and cattle, respectively [22]. The country incurs one of the highest burdens of livestock rabies in the world, with annual national economic loss from cattle rabies estimated between $209 million [23] and $412 million [3]. Accurate information on the incidences of rabies in humans, dogs and livestock is, however, scarce due to the absence of proper registration systems [21], but numbers are expected to be higher as many cases are not reported [24].

In the four zones of Gondar (Central, West, North and South Gondar), Northwest Ethiopia, University of Gondar and Ohio State University were vaccinating dogs for the last five years targeting to control rabies outbreaks. However, the realized vaccination coverage was lower than the recommendation by WHO in controlling rabies. One reason for the low coverage was, except few owners in urban areas, large number of dog owners were not willing to vaccinate their dogs against rabies at their own costs. This problem of dog owners was also reported by Beyene et al. [25] in other parts of Ethiopia. This is a challenge in controlling rabies using vaccination in Ethiopia. However, as Wera et al. [14] reported from Indonesia, money may not be the only factor that motivates owners in their decisions to vaccinate their dogs. Non-monetary factors, such as dog owners’ characteristics and psychological factors such as attitude (AT), subjective norms (SN) and perceived behavioral control (PBC) also influence the motivation of dog owners to vaccinate their dogs. Understanding the drivers of dog owners’ motivation to participate in rabies control measures [14] and their willingness to pay (WTP) for rabies vaccine [26], therefore, are essential for the implementation of effective dog vaccination campaign. To our knowledge, no studies have investigated factors motivating dog owners to improve rabies control in Ethiopia or other sub Saharan countries. More evidence about dog owners’ acceptance and WTP for rabies vaccine is essential to evaluate the feasibility of implementation of vaccination programs when the vaccine is available and also to provide insights into future pricing considerations and demand forecasts. The present study was conducted to assess the intention of dog owners to control rabies, their WTP for rabies vaccine, and factors influencing their intention and WTP in northwestern Ethiopia.

## Materials and methods

### Ethics statement

The study was ethically reviewed and approved by Ethical Clearance Committee of University of Gondar, Ref. no. VP/RTT/05/1037/2022, Date: 27, July 2022. Informed verbal consent was obtained from dog owners who were volunteered to participate in the study and all data were analyzed and reported anonymously.

### Study area

**Fig 1:**
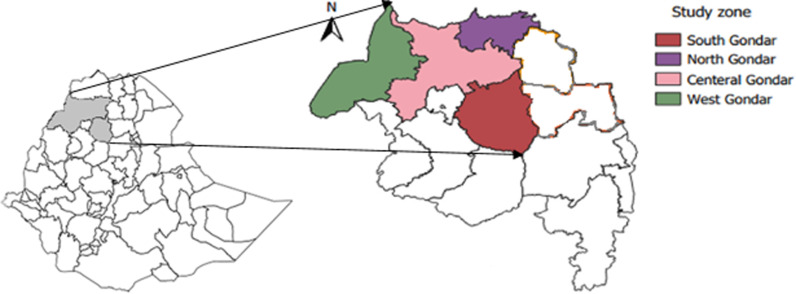
Map of the study area (shaded in gray).

The study was conducted in northwestern Ethiopia. The area is selected because of the high prevalence of animal and human rabies [21]. The area covers four zones: namely West Gondar, North Gondar, Central Gondar, and South Gondar zones. The study area is bordering Sudan in the west, Benshangul Gumuz region in South west, North and East Gojam Zones in South, North Wollo and Waghemra zones in the East, and Tigray region in North (Fig 1).

The human population was 328,006 in West Gondar; 2,551,158 in South Gondar; 3,267,254 in Central Gondar and 1,176,412 in North Gondar zones [27]. According to the local veterinary offices, the population of dogs in the study area was estimated to be 1639 in West Gondar, 2090 in Central Gondar, 1945 in North Gondar, and 2531 in South Gondar zones.

### Theory of planned behavior

**Fig 2:**
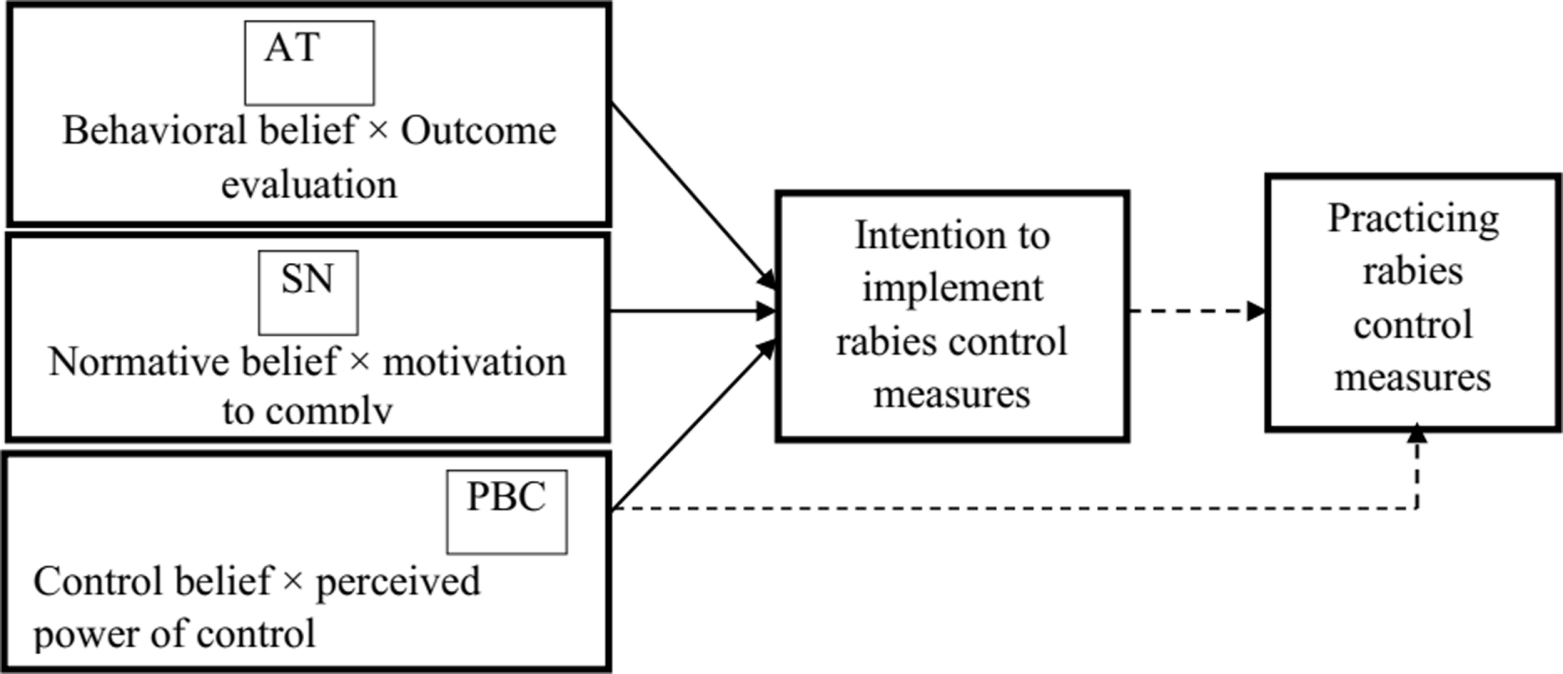
Framework for dog owners’ intention to participate in rabies control measures (taken from Ajzen, 1991). AT = Attitude, SN = Subjective norm, PBC = Perceived behavioral control. Dotted lines indicate associations that are not studied in this paper.

According to the TPB, a person’s intention to perform (not perform) a behavior can be predicated with high accuracy from his AT, SN, and PBC [28] (Fig 2). A positive AT indicates for instance that dog owners believe that a particular control measure is beneficial in reducing rabies; this positive AT may be a reason to participate in rabies control measure. Positive SN indicates that the opinions of family members, neighbors, animal and public health authorities, and local community could influence the decision of the dog owner to participate in a certain rabies control measure. Perceived behavioral control concerns that the dog owners’ beliefs about their resources and ability to perform certain rabies control measure, such as time, money, and ability to catch and restrain dogs. The beliefs of dog owners about their resources could influence their intention to participate in a rabies control measure. The theory of planned behavior (TPB) framework has been used in several studies to obtain insight in the psychological factors that influence intentions related to rabies control. This approach has, for instance, been used by Thomas et al. [29] and Wera et al. [14] to investigate dog owners’ participation in rabies control.

### The contingent valuation method

Contingent valuation method (CVM) is a survey-based technique for eliciting preferences for non-market valuation method in a form which allows one to estimate how survey respondents’ trade-off private consumption in monetary terms to stated hypothetical market prices [30]. Different hypothetical market scenarios were created for rabies vaccine. A double bound dichotomous choice method bid design techniques was used. Survey respondents were asked to state their response to the hypothetical market scenarios. The data collected by such surveys was then analyzed in a similar manner as the choices made by consumers in actual markets [30]. The options range of price is based on the approximate minimum–maximum price range of rabies vaccines in Ethiopia. Contingent evaluation method has been used in several willingness to pay (WTP) studies in animal disease control [31–35] including rabies vaccination [26].

### Questionnaire design

In construction the questionnaire, first potential rabies control measures applicable according to northwestern Ethiopian circumstances were identified. The questionnaire consisted of introductory statements explaining the purpose and importance of controlling rabies and vaccination of dogs and comprised of sets of questions that aimed to explore intention of controlling rabies and determinants of intention.

Two statements were aimed at asking the intentions towards vaccination control and one statement was intended about intentions towards control of rabies by leashing dogs. Behavioral, normative, and control beliefs that can affect dog owners’ behavior and corresponding evaluations were identified based on our experience. These latent variables were measured by asking respondents about statements concerning their beliefs and the corresponding evaluations.

The behavioral beliefs of AT (bbAT) towards the intention to implement dog vaccination were measured by three items referring to their impact in controlling rabies: reducing incidence, preventing transmission and spreading between dogs or other animals. To measure the bbAT towards leashing dogs were measured by three items referring to the impact of leashing dogs on controlling rabies: the effect of leashing dogs in preventing dogs from rabies infection, preventing rabies transmission between dogs and from dog to human. Corresponding outcome evaluations of the behavioral beliefs were measured by statements asking dog owner to give his own positive or negative judgments about rabies control by using vaccination and leashing dogs.

To measure the SNs, veterinarians, health extension technicians, family members and agricultural extension workers were considered most important to dog owners to approve or disapprove rabies control measures. Normative beliefs were evaluated by statements asking the dog owner about the opinion of these five groups of people to approve or disapprove rabies control measures. Motivations to comply were measured by statements asking the dog owner whether the opinion of those people influences his intention to implement rabies control measures.

Perceived behavioral control was evaluated in relation to the relative ease or difficulty involved in undertaking rabies control measures. Perceived behavioral control beliefs were measured by statements evaluating whether rabies control measures were difficult to implement, were time consuming and were considered expensive by the dog owner. The corresponding perceived power of control was measured by statements evaluating how confident a dog owner feels about being able to implement, having time to implement and having money to cover the costs to implement a rabies control measure.

As behavioral beliefs of attitudes towards vaccination of dogs were measured by asking respondents about the importance of vaccination in controlling rabies, it is unlikely respondents’ would have different responses. However, they differ when it comes to whether the vaccination is free of charge or with charge. Therefore, no separate questions asked for attitudes about vaccinating dogs with fair price and vaccinating dogs with free of charge, i.e., respondents were asked attitude questions about vaccinations once. We believe that respondents had power of control to vaccinate their dogs free of charge; therefore, we did not ask respondents about PBC for free of charge.

Dog owners were asked to state their level of agreement for each statement on a 7 point Likert’s scale. If the statements referring to beliefs were evaluated using a unipolar Likert scale (1 to 7) or bipolar 7 point Likert scale (−3 to 3), statements referring to outcome evaluations, motivation to comply and perceived power of control questions were evaluated using a bipolar 7 point Likert scale (−3 to 3) or unipolar Likert scale (1 to 7). Different groups of people can have experiences and information that differ in important ways from the experiences of others [36,37]. Therefore, additional data were collected about demographic factors expected to influence behavioral, normative, or control beliefs of dog owners.

In the WTP survey, a particular elicitation method, a payment or bidding game [38] was used with a series of yes/no questions that aim to find the maximum WTP. During questionnaire administration, the first bid amounts were distributed equally and randomly among respondents. Then, the first bid questions were followed up by second bid questions with bid amount of  ±  25% the initial bid amounts depending on the response to the first bid amount. If the response to the initial bid amount was ‘yes’ the follow up bid amount would increase by 25% and if the response to the initial bid amount was ‘no’ the follow up bid amount would decrease by 25%.

The initial bid amounts were 20, 30, 40 and 50 Ethiopian Birr; presented in Table 1. The stated price set was proposed based on the current price range of rabies vaccines in Ethiopia as we got from respective animal health offices in the selected study area. In this study, dog owners were made to bid for a hypothetical vaccine with protection effectiveness of almost reaches 100% when first shot given 3 months and above, first booster dose at 1.5 years and additional boosters every 3 years by local veterinary officials.

**Table 1:** The double-bound dichotomous choice questionnaire bid structure.

No	First bid (administered randomly one for each respondent) (ETB^1^)	Answer for first bid	Second bid (ETB)
1	20	no	15
		yes	25
2	30	no	22.5
		yes	37.5
3	40	no	30
		yes	50
4	50	no	37.5
		yes	62.5

^1^Ethiopian Birr

The questionnaire was prepared in English before it was translated to local language (Amharic) and back to English to check consistency of the questions. The final questionnaire was administered by face-to-face interviews. The English version of the whole questionnaire is available in supporting information file (S1 Table and S1 Appendix).

### Sample size estimation

The required sample size to estimate the proportion of dog owners’ intention and perception about dog rabies control and WTP for rabies vaccine were based on 50% occurrence of a state or condition, 5% percentage maximum error required, Z =1.96 value for 95% confidence intervals [39].


$$n=\frac{P\left(100-P\right)Z^{2}}{E^{2}}$$

Where:

n = the required sample size.

P = the percentage occurrence of a state or condition.

E = the percentage maximum error required.

Z = the value corresponding to level of confidence required.

A sample size of 384 dog owners’ calculated. Expecting 10% incomplete interviews, the sample size for the two studies was determined to be 423 dog owners.

### Sampling technique

Using cross-sectional design, data was collected from each of selected dog owner one instance from October 2022 to March 2023. During the sampling, districts and towns: Fogera and Debre Tabor from South Gondar, Genda-Wuha and Metema from West Gondar, East Dembia and Tach Armachiho from Central Gondar, and Debark from North Gondar zones were selected based on our judgment of representativeness and occurrence of rabies. Two Kebeles (the smallest administrative unit in Ethiopia) were selected from each of the six districts based on accessibility. Heads of dog owned households were the sampling units. Then list of dog owners was created in each Kebele based on knowledge of informants, veterinary technicians working in the nearby veterinary clinics and persons working in Kebeles and random numbers were assigned for each dog owners. Dog owners were approached for interview based on the order of assigned random numbers for the four different respondent groups created based on the initial bid amounts: 20, 30, 40 and 50 Ethiopian Birr. When a dog owner was not willing to be interviewed, he was skipped and the dog owner with the subsequent number on the list was approached. In that way 423 dog owners who were volunteered to participate in the study were included from the total list of 3,328 dog owners.

### Data management and analysis

Accuracy of the data was checked to minimize data entry error. The score given for each of the behavioral, normative and control belief statements was multiplied by the corresponding outcome evaluation, motivation to comply, or perceived power of control, respectively, to create a new variable that represented the product score. In this way, product composites were created for the three TPB factors (AT, SN and PBC). Cronbach’s alpha was calculated among the product composites of AT, of SN and of PBC to test internal consistency. The product composites were considered to have internal consistency and, therefore, measure similar construct, if Cronbach’s alpha was  ≥  0.7 [40]. In that case, product composites were averaged to obtain one single measure (mean score) for AT, SN and PBC (Eqs 1–3). For those constructs where the Cronbach’s alpha was < 0.7, product composites were used as separate TPB factors.


$$AT=\frac{\sum_{i=1}^{n}(bbi*oei)}{n}$$ (1)

Where i = the product composite item i and n = number of items for AT, bb = behavioral belief, oe = outcome evaluation.


$$SN=\frac{\sum_{i=1}^{n}\left(nb_{i}*mc_{i}\right)}{n}$$ (2)

Where i = the product composite item i and n = number of important referents for SN, nb = normative belief, mc= motivation to comply.


$$PBC=\frac{\sum_{i=1}^{n}\left(cb_{i}*ppc_{i}\right)}{n}$$ (3)

Were i = the product composite item i and n = number of relevant control belief items, cb = control belief, ppc = perceived power of control.

The TBP factors are classified into three categories based on the distribution of the product composites as weak AT, weak SN and weak PBC (product composite  ≤  0); moderate AT, moderate SN and moderate PBC (> 0 to < 18); and strongly positive AT, strongly positive SN and strongly positive PBC (≥ 18). A dependent variable was constructed for the intention towards each of the intention of rabies control strategies by classifying dog owners into low intenders (Likert score for intention ≤ 0) versus high intenders (Likert score for intention > 0). The categorized intention variables were used as dependent variable in logistic regression models (one model for each intention) with AT, SN and PBC as predictors. As is commonly done in TBC studies, the full models were presented. The effect of background factors on TPB variables were analyzed by using logistic regression models with TPB factors (AT, SN and PBC) as a dependent variables.

Interval regression analysis was used to estimate the mean dog owner’s WTP for rabies vaccine and to identify potential factors that influence dog owners’ WTP. Four binary response for double bound dichotomous contingent evaluation question: dog owners were WTP neither the initial bid amount nor the lower bid amount (“no”, “no”), were not WTP for the initial amount but accepted the lower bid amount (“no”, “yes”), were WTP for the initial bid amount but not for the higher follow up amount (“yes”, “no”), and the last response was accepting both the initial and the higher follow up bid amount (“yes”, “yes”). These create four possible intervals and three types of censoring namely: left censoring, interval censoring and right censoring. In the interval regression modeling, stepwise regression analysis was used and significant variables were retained. Multicolinearity was checked using both variance inflation factor and pairwise correlation analysis. The predictor variable having greater than value 10 variance inflation factors was considered presence of co-linearity. The final model was presented by elimination of non-significant variables (p > 0.05). Confounder was checked using the model building process. The level of significance was set a prior at P < 0.05 and Stata release14 (StataCorp LLC, USA) was used in all the data analysis.

## Results

### Descriptive statistics

**Fig 3:**
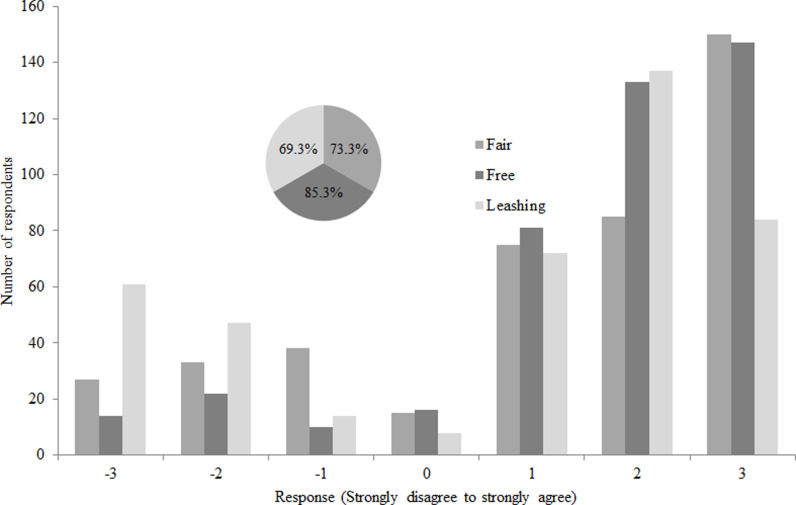
Dog owners’ intentions to implement rabies control measures in North-Western Ethiopia.

Majority of dog owners had a positive intention to implement rabies control measures. Larger number of dog owners (85.3%) intended to participate in the rabies vaccination campaign if the government would provide the vaccine free of charge. A large number of dog owners had a positive intention to participate in the rabies vaccination campaign if the cost of vaccination is fair (73.3%), while the lowest proportion of dog owners had a positive intention to control rabies by leashing dogs (Fig 3).

In general, dog owners had positive AT, positive SN and positive PBC to implement the stated rabies control measures. With respect to PBC, all of the dog owners (100%) had time for leashing dogs, but they had, relatively, lower percentage of AT and SN (Table 2).

**Table 2:** Descriptive statistics of attitude (AT), subjective norms (SN) and perceived behavioral control (PBC) measured with respect to the rabies control measure intentions in 423 dog owners in North-Western Ethiopia.

Rabies control measures	Variables	Cronbanch alpha	Weak (n)	Moderate (n)	Strong positive (n)	Response (%)
**Vaccination of dog with fair price of vaccine or free of charge**	AT(bb*oe)	0.7695	103	283	37	320 (76)
**Vaccination of dog with fair price of vaccine**	SN (nb*mc)	0.7595	136	187	100	287 (68)
	PBC (cb*ppc)	0.8938	132	195	96	293 (69)
**Vaccination of dog free of charge**	SN_1_ (nb*mc)	0.5924	61	279	83	362 (86)
	SN_2_ (nb*mc)	0.5533	56	305	62	367 (87)
	SN_3_ (nb*mc)	0.5373	57	293	73	366 (86.5)
	SN_4_ (nb*mc)	0.7106	95	281	47	328 (77.5)
**Leashing of dog**	AT(bb*oe)	0.8939	127	202	94	296 (70)
	SN (nb*mc)	0.9063	142	186	95	281 (66)
	PBC_1_(cb*ppc)	0.146	155	242	26	268 (63)
	PBC_2_(cb*ppc)	0.7622		378	45	423 (100)
	PBC_3_(cb*ppc)	0.0898	157	237	29	266 (63)

AT = Attitude, bb = behavioral beliefs, oe = outcome evaluation, SN = subjective norm, nb = normative belief, mc = motivation to comply, PBC = perceived behavioral control, cb = control belief, ppc = perceived power of control, 1–4 = the 1^st^, the 2^nd^, the 3^rd^ and the 4^th^ items of SN and PBC that Cronbach’s alpha value < 0.7 and used as separate variables for vaccination of dog free of charge and leashing of dog.

A total of 447 respondents were included in the willingness to pay survey. Data of 24 respondents were not included in the analysis because of unwanted or incomplete response. Majority of respondents (58.16%) were males. About 74.23% of the respondents were farmers followed by private workers (merchants) (21.99%). About 228 dogs were vaccinated within the last two years. Forty five percent of the respondents were low-income group, < 2001 ETB per month. Almost fifty three percent of the dog owners stated that they have vaccinated their dogs once within the past two years. Detail of the socioeconomic variables is presented in Table 3.

**Table 3:** Summary of socio demographic and other factors possible to influence dog owners’ willingness to pay for rabies vaccine (n=423).

Variables	Levels	Frequency (%)
**Age of respondents’ in years**	18–35	73 (17.3)
	36–50	296 (69.98)
	> 50	54 (12.77)
**Vaccinating dogs in the last two years**	Yes	228 (53.9)
	No	195 (46.1)
**Household monthly income in Ethiopian Birr**	≤ 2000	191 (45.15)
	> 2000 to < 10000	151 (35.7)
	10000 to15000	51 (12.06)
	>15000	30 (7.09)
**Number of persons per household**	1–3 person	195 (46.1)
	4–6 person	81 (19.15)
	> 6 person	147 (34.75)
**Job status of respondents**	Farmer	314 (74.23)
	Merchant	93 (21.99)
	Gov.t servant	16 (3.78)
**Age of dog**	6 month–1 year	130 (30.73)
	1 year–2 years	132 (31.21)
	> 2 years	161 (38.06)
**Gender of respondents**	Male	246 (58.16)
	Female	177 (41.84)
**District**	Woreta	96 (22.70)
	Debretabour Town	67 (15.84)
	Metema	101 (23.88)
	Dembia	75 (17.73)
	Debark	45 (10.64)
	Gondar	39 (9.22)
**Sex of dog**	Male	242 (57)
	Female	181 (43)

Overall 423 respondents were answered the double bounded dichotomous question and 263 (62%) of them accepted the first bids whereas 142 (54%) of them refused to for the second higher bid amount offered to them for rabies vaccine (Table 4). Among the respondents refused the initial bid amount, 136 (85%) of them accepted the second lower bid amount. The willingness to pay for the stated vaccine price amount was observed to decrease with a second higher bid amount. The percentage of WTP (‘yes’ responses) for the first bids of 20 ETB, 30 ETB, 40 ETB and 50 ETB were 81%, 57%, 49% and 50% respectively (Table 4).

**Table 4:** Summary of data of respondents (N = 423) in the double dichotomous contingent valuation willing to pay survey for rabies vaccine.

First bid in ETB	First bid response		Follow up price amount	Follow up price response	
	Response	No. of response (%)	No/no response (%)	No/ yes response (%)	
**20**	No	27 (19)	15	11 (41)	16 (59)
	Yes	115 (81)	25	68 (59)	47 (41)
**30**	No	47 (43)	23	44 (94)	3 (6)
	Yes	63 (57)	38	36 (57)	27 (43)
**40**	No	41 (51)	30	8 (20)	33 (80)
	Yes	40 (49)	50	16 (40)	24 (60)
**50**	No	45 (50)	38	2 (4)	43 (96)
	Yes	45 (50)	63	22 (49)	23 (51)
**Over All**	No	160 (38)	166 (39)	24 (15)	136 (85)
	Yes	263(62)	157 (61)	142 (54)	121(46)

### Theory of planned behaviour factors associated with intentions to control rabies

Attitude was statistically significantly associated (P < 0.05) with the intention to implement rabies control measure by vaccination of dogs with fair price, vaccination with free of charge and leashing of dogs. Perceived behavioral control was significantly associated with the intention towards leashing of dogs but not significantly associated to vaccination with fair charge amount (Table 5).

**Table 5:** Statistical associations between, subjective norms and perceived behavioral control, and the intentions to control rabies.

Intention of rabies control	Variables	Weak	Moderate OR (95%CI)	Strong positive OR (95%CI)
**Vaccination with fair price or free of charge**	AT(bb*oe)	Ref.	**20.8 (10.4- 41.5)**	**157.0 (19.1**–**1289.3)**
**Vaccination with fair price**	SN (nb*mc)	“	1.8(0.9–3.3)	**15.63 (5.5**–**44.3)**
	PBC(cb*ppc)	“	1.2 (0.6–2.6)	0.7 (0.3–1.5)
**Vaccination of dog free of charge**	SN_1_ (nb*mc)	“	**8.1 (1.9–34.6)**	3.8 (0.8–17.6)
	SN_2_ (nb*mc)	“	**31.2 (7.0–139.1)**	**44.7 (6.7–298.6)**
	SN_3_(nb*mc)	“	0.9 (0.2–4.9)	5.2 (0.6–43.2)
	SN_4_(nb*mc)	“	0.1 (0.03–0.6)	7.0. (0.5–93.6)
**Leashing of dogs**	AT(bb*oe)	“	**6.1 (3.3–11.1)**	**15.4 (6.7–35.1)**
	SN (nb*mc)	“	**3.9 (2.1–7.2)**	1.4 (0.7–2.8)
	PBC_1_(cb*ppc)	“	**8.2 (3.3–20.2)**	**13.0 (3.0–56.9)**
	PBC_2_(cb*ppc)	–	Ref.	0.9 (0.4–2.3)
	PBC_3_(cb*ppc)	Ref.	0.4 (0.2–0.9)	0.3 (0.1–1.6)

AT = Attitude, bb = behavioral beliefs, oe = outcome evaluation, SN = subjective norm, nb = normative belief, mc = motivation to comply, PBC = perceived behavioral control, cb = control belief, ppc = perceived power of control, 1–4 = the 1^st^, the 2^nd^, the 3^rd^ and the 4^th^ items of SN and PBC that Cronbach’s alpha value < 0.7 and used as separate variables for vaccination of dog free of charge and leashing of dog.

### Background factors associated with TPB factors and willingness to pay for anti-rabies vaccine

Among background factors tested age was statistically significantly associated with the PBC to vaccinate dogs with fair vaccine cost while age and sex were statistically significantly associated (P < 0.10) with the attitude of leashing of dogs (Table 6).

**Table 6:** Statistical association (P < 0.10) between background factors and dog owners’ attitude (AT), subjective norms (SN) and perceived behavioral control (PBC) that were significantly associated with intentions to implement rabies control measures.

Rabies control measure	TBP factor	Background factor (P < 0.1)	Level	OR^1^ (95% CI^2^)
**Dog Vaccination with fair vaccine cost**	AT	Age	Female	Ref^3^.
			Male	0.7 (0.4–1.1)
	SN	Education background	Non educated	Ref.
			< Grade 8	1.7 (0.9–2.8
			≥ grade 8	0.9 (0.5–1.5)
	SN	Religion	Orthodox	Ref.
			Muslim	1.4 (0.8–2.6)
			Others	2.3 (0.9–5.4)
	PBC	Age	age ≤ 45	Ref.
			Age > 45	**1.7 (1.2–2.5)**
**Dog vaccination with free of charge**	SN	Sex	Female	Ref.
			Male	1.2 (0.6–2.3)
**Leashing of dogs**	AT	Education background	Non educated	Ref
			< Grade 8	0.5 (0.3–0.8)
			≥ grade	0.6 (0.3–1.1)
		Sex	Female	Ref.
			Male	**1.8 (1.2–2.9)**
		Age	Age ≤ 45	Ref.
			Age > 45	**0.4 (0.3–0.7)**
	PBC1	religion	Orthodox	Ref.
			Muslim	1.4 (0.8–2.7)
			Others	3.5 (0.9–13.5)

^1^Odds ratio.

^2^95% confidence interval.

^3^Reference category

Eight of the variables were associated, in univariable analysis, with dog owners WTP for rabies vaccine. However, only three of the variables were significantly associated (P < 0.05) in the multivariable interval regression analyses (Table 7). All the data in this paper are available in supporting information file (S2 and S3 Tables).

**Table 7:** Summary of factors associated with willingness to pay (P < 0.05) for rabies vaccination in North West Ethiopia.

Variables	level	Estimate	P value	95% CI^1^
**Dog owner income in ETB** ^2^	≤ 2,000	Ref^3^.		
	> 2,000 to < 10,000	2.78	0.10	−0.54 – 6.11
	10,000–15,000	10.65	0.01	5.23 – 16.06
	> 15,000	12.31	0.01	5.28 – 19.34
**Job status of respondents**	Farmer	Ref.		
	Government service	4.39	0.29	−3.77 – 12.55
	Merchant	6.14	0.02	2.32 – 9.95
**Vaccinating dogs in the last two years**	Yes	Ref.		
	No	−5.61	0.01	−8.67 – −2.56

^1^95% confidence interval.

^2^Reference category.

^3^Etiopian birr.

## Discussion

### Limitation of the study

We have used TPB to explore the intention of dog owners towards implementing rabies control measures, and contingent evaluation method to evaluate WTP for rabies vaccine. Theory of planned behavior assumes that behavior is determined by a person’s attitudes, subjective norms, and perceived behavioral control. It overlooks external factors, lack of consideration for emotions, and potential discrepancies between intention and action [41,42]. Depending on data collection methods, there are numerous approaches and corresponding analytical techniques for measuring WTP. It can utilize actual or simulated price response data (revealed preference) or survey technique (stated preference). In the survey-based techniques, there exist direct and indirect surveys for collecting the relevant data [43]. The data for the current study was collected by stated preference techniques of direct surveys, respondents are asked to state their WTP to hypothetical bid questions. Data collected in direct surveys in stated preference approach has limitations. One of the challenges is that respondents may overstate prices because of prestige effects or understate prices because of collaboration effects [44]. They may also attempt to quote artificially lower prices, since many of them perceive their role as conscientious buyers as that of helping to keep prices down [45]. Therefore, these issues may affect the validity of the estimation.

### Intention of dog owners to control rabies

This study was conducted to assess the intention of dog owners to implement rabies control measures, their WTP for rabies vaccine, and determinants of intention and WTP of dog owners in northwestern Ethiopia. The study was conducted based on theoretical planed behavior conceptual framework to explore intention of dog owners towards three rabies control measures. We found that majority of dog owners (85.3%) intended to participate in the rabies vaccination campaign if the government would provide the vaccine free of charge. It was not possible to compare this finding with similar studies, as there is no, to our knowledge, previous studies on intention of dog owners in rabies vaccination in Ethiopia. However, vaccination fee is one reason for dog owners not to join a vaccination campaign [46]. Thomas *et al*. [29] from South America and Wera *et al*. [14] from Indonesia reported that 100% and 96% of dog owners, respectively, intended to vaccinate their dogs when vaccination was given free of charge. If the government provides vaccinations for dogs with fair price, the intention to vaccinate decreased to some extent (73.3%) and these measures represents the main rabies control measure currently applied in North West Ethiopia, in which the dog vaccination is subsidized. Transport costs and daily allowance of persons that participate in the dog vaccination are covered by the government; i.e., only direct costs related to the vaccination are covered by the dog owner. The reason for decreasing intention when dog owners are charged for vaccination may be due to the vaccination cost which affects participation of few dog owners on vaccination campaign. Approximately, 70% of dog owners had positive intention to leash their dogs when rabies outbreak occurs within their village.

Dog owners who had positive attitude had higher odds of intention to implement rabies control measure by vaccination of dogs with fair price. This finding agrees with the report of Ebuy et al. [47] and Hagos et al. [48] in Tigray region, Ethiopia, who described control of rabies through vaccination was significantly associated with positive dog owners’ attitude. As the attitude variables were often significantly associated with intention to participate in a rabies control measure, rabies educational campaign focusing on the benefit of rabies control measures is expected to increase the intention of dog owners to participate in future rabies control measures. Similarly dog owner having moderate and strong positive attitude had, respectively, 6.1 and 15.4 higher odds of intention than dog owners who had weak intention to control rabies by leashing of dogs. This finding is consistent with the report of Serebe et al. [49] in North West Ethiopia. In that report good attitude of dog owners had significant association to the intention towards implementing rabies control by confining dogs into their house. Subjective norm for dog owners was explored with statements about the importance of family members, neighbors, the veterinarian, and health extension and development extension workers. Dog owners who had a strong positive SN had higher odds of intention in controlling rabies through vaccination when rabies vaccine is given with fair price. This finding is in line with the study report by Beyene et al. [25].

It is not surprising to find that dog owners who had positive PBC had higher odds of intention to control rabies by vaccination than dog owners who had weak PBC. Because a dog owner who has the skill in handling dogs, who has time and money to cover cost of vaccination is likely to vaccinate his dog. Even though, the situation was under different circumstances, Wera et al. [46,50] reported similar findings.

Dog owners who had education level of grade eight and above had lower attitude to control rabies by vaccination compared to that of owners who did not have education. This finding is in line with Gebrezgiher et al. [51] from Northern Ethiopia. This contradicts what we know from the literature. It is likely that dog owners who have had more education feel more confident and had lower attitude and ignorance behavior for advice given by veterinary officials. Dog owners who were above 36 years of age had higher positive PBC and higher odds of intention to control rabies by vaccinating dogs with fair price than dog owners who had weak PBC. The finding in the current study was in line with the report of Gebrezgiher et al. [51]. The intention to control rabies by vaccinating dogs increases with age of the owners, the explanation for this might be that dog owners may create money to cover the price for vaccine, develop skills how dogs are tied up and confined during vaccination against rabies and likely to have family to take the dog to vaccination campaign.

### Willingness to pay for rabies vaccine

This study was conducted to assess dog owners’ willingness to pay for rabies vaccine and to identify socio demographic factors that influence dog owners’ WTP for rabies vaccine in North West part of Ethiopia. We found that the majority of dog owners (62%) were WTP for the suggested price bid amounts, in general, for rabies vaccine. The finding in the current study was in line with the work of Birhane et al. [26] who reported that majority of dog owners were WTP for dog vaccination. This is expected in Northwest Ethiopia where the access to dog vaccine is limited while the zoonotic importance and severity of rabies are known by dog owners. However, interestingly, the proportion of dog owners WTP for the vaccine has shown great variability when it comes to specific price bid amounts. Greatest majority of dog owners (81%) were WTP the initial price bid amount (20 ETB). The percentage of dog owners WTP for the vaccine decreased from 81% to 44% when the bid amount increased by 25% from the initial bid price, and it further decreased to 31% when the bid amount increased to 50 ETB. This may reflect that dog owners had multiple price bid amounts in mind and their WTP varies on the cost of vaccine. Apparently, dog owners are WTP “certain price” for rabies vaccine, which is quite an intangible, but their WTP is variable when the price become more tangible. The reason for this could be related to financial resources to pay the vaccination fee as well as the cost of rabies vaccine [46,52]. Costs of rabies vaccine for dogs vary widely according to the producing campaigns. A summary of 10 published studies had shown that, globally, mass dog vaccination costs per dog vary from US$ 1.56 – US$ 11.33 with average costs of US$ 4.03 [5]. Such cost is expensive which supports that, in the current study, income was statistically significantly associated with WTP for vaccination to control rabies; dog owners with higher monthly income had higher odds of WTP to cover costs of vaccination for their dogs to control rabies. When the general WTP percentage (62%) calculated from the response of dog owners for the four price bid amounts, only dog owners who are not WTP for higher price bid amounts disagree with the statement asking WTP for higher price bid amounts.

The mean WTP for the vaccine as estimated using the interval regression model parameterized from the double-bound dichotomous questionnaire data were ETB 59.25 (1.14 USD) per year. This finding agrees with the report of Andrew et al. [53] in Addis Ababa, Ethiopia. This WTP estimate is significantly higher than the 20 Birr/ 25 Birr per dose for annual vaccination charged currently for vaccine produced by national veterinary institute in Ethiopia. This higher level of WTP for the vaccine may indicate possibility of outsourcing rabies vaccination to private sector as the difference in the market price and WTP may cover vaccine administration cost.

Willingness to pay for rabies vaccination decreased in dog owners who did not vaccinate dogs against rabies in the last two years. This can be explained in relation to lack of awareness of dog owners about the risk of rabies and perceived lack of evidence of effectiveness of the vaccine. This suggests need of increasing awareness on risk and severity of rabies, and importance and implementation of anti-rabies dog vaccination. It is not surprising that dog owners who earn higher monthly income had higher odds of WTP for rabies vaccination because they can afford the cost of vaccination if they perceive rabies is a dangerous disease. There was difference in dog owners’ WTP for vaccinating dogs in controlling rabies based on their job type. This finding was in line with the report of Gebrezgiher et al. [51] from Laelay Machew district, north Ethiopia and Ebuy et al. [47] in South east Tigray. This could be related to the difference in dog owners’ income. Dog owners working as merchants, in general, have higher income than dog owners working as farmers. However, we did not find confounding between job type and income of dog owners.

Proactive and sustainable vaccination programs have proven their efficacy in the eradication of domestic dog rabies which motivates the fight against rabies control. The results of the current study, in general, have shown that dog owners were willing to contribute and pay for rabies vaccine to receive immediate vaccination prior to rabies outbreak. This information can be used by veterinary service and public health agencies when planning dog vaccination programs in controlling rabies and to policy makers in their decision to subsidize rabies vaccination.

## Conclusion

Male dog owners and dog owners belonging to lower age groups had significantly higher AT towards leashing dogs: age > 45 years decreased AT and being male dog owner increase AT for leashing of dogs. Although the WTP for rabies vaccine has shown variability between specific price bid amounts, in general, majority of dog owners were WTP for the suggested price bid amounts. Increasing the intention of dog owners to implement rabies control interventions directed at changing their AT and their SN are expected to give the largest improvement in rabies control behavior and provisions of anti-rabies dog vaccine with lower price improve participation of dog owners in anti-rabies vaccination campaign.

## Supporting information

S1 Table Questionnaire - intention of dog owners. (DOCX)

S1 Appendix Questionnaire - willing to pay of dog owners. (DOCX)

S2 Table Data - willing to pay of dog owners. (XLSX)

S3 Table Data - intention of dog owners. (XLSX)
