# Supplementary material for: Dog owners’ intention to control rabies and their willingness to pay for rabies vaccine in Northwestern Ethiopia
Source: PLOS Glob Public Health. 2025 Mar 11;5(3):e0003974. doi: 10.1371/journal.pgph.0003974 (PMC11896628; doi:10.1371/journal.pgph.0003974)
Supplement: S1 Table — (DOCX) [file pgph.0003974.s001.docx]

**Questionnaire on attitudes, subjective norms, perceived behavioral control and intentions of dog owners to control rabies**

Dear respondent,

The purpose of this questionnaire is to obtain data for the research “Dog owners’ intention to control rabies and their willingness to pay for rabies vaccine in northwestern Ethiopia” using the Theory of Planned Behavior. The aims of the study were to assess the intention of dog owners to control rabies, and to identify factors influencing their intention in northwestern Ethiopia.

Your response is important for the success of the study. So, I kindly request your willingness to answer questions I am going to ask you. Your response will be confidential; I assure you that the data will be analyzed and reported anonymously.

Please be aware that you have the right to stop or withdraw from the study with any reason.

Thank you in advance for your willingness to participate in the study.

| **Intention of Dog Owners** |  |
| --- | --- |
| If the government recently provided **vaccination** for dogs to prevent rabies with **affordable/fair price**, would you vaccinate your dog? | Strongly disagree -3 -2 -1 0 1 2 3 Strongly agree |
| If the government recently provided **free vaccination** to dogs to prevent rabies, would you vaccinate your dog? | Strongly disagree -3 -2 -1 0 1 2 3 Strongly agree |
| **Attitude For Vaccination of Dog With Faire Price** |  |
| **Behavioural Beliefs** |  |
| Vaccination a dog reduce the incidence of rabies | Strongly disagree 1 2 3 4 5 6 7 Strongly agree |
| vaccinating a dog prevents rabies transmission from dog to person | Strongly disagree 1 2 3 4 5 6 7 Strongly agree |
| Vaccinating a dog prevent rabies from spreading between dogs or other animals? | Strongly disagree 1 2 3 4 5 6 7 Strongly agree |
| **Outcome Evaluation** |  |
| dog vaccination important to reduce the incidence of rabies | Very unimportant -3 -2 -1 0 1 2 3 Very important |
| Vaccination dog is important to reduce transmission from dog to man | Very unimportant -3 -2 -1 0 1 2 3 Very important |
| Vaccination of dog against rabies important to prevent dog rabies from being transmitted between dogs or other animals | Very unimportant -3 -2 -1 0 1 2 3 Very important |

| **Subjective Norm For Vaccinating Dog With Fair Charge** |  |
| --- | --- |
| **Strength of Normative Belief** |  |
| What do you think the following people know about rabies prevention? |  |
| Veterinarians think that control of rabies is | Very unimportant -3 -2 -1 0 1 2 3 Very important |
| The health extension technicians thinks that control of rabies is | Very unimportant -3 -2 -1 0 1 2 3 Very important |
| My family think that control of rabies is | Very unimportant -3 -2 -1 0 1 2 3 Very important |
| The agricultural technicians think that control of rabies is | Very unimportant -3 -2 -1 0 1 2 3 Very important |
| **Motivation to Comply** |  |
| Does the opinion of the following people regarding reduction of rabies influence your intention to implement preventive measures? |  |
| Veterinarians | Not at all 1 2 3 4 5 6 7 Very much |
| The health extension technician | Not at all 1 2 3 4 5 6 7 Very much |
| My family | Not at all 1 2 3 4 5 6 7 Very much |
| The agricultural technicians | Not at all 1 2 3 4 5 6 7 Very much |
| Other dairy farmers | Not at all 1 2 3 4 5 6 7 Very much |

| **Subjective norm for vaccinating dog with free of charge** |  |
| --- | --- |
| **Strength of normative belief** |  |
| What do you think the following people know about rabies prevention? |  |
| Veterinarians think that control of rabies is | Very unimportant -3 -2 -1 0 1 2 3 Very important |
| The health extension technicians thinks that control of rabies is | Very unimportant -3 -2 -1 0 1 2 3 Very important |
| My family think that control of rabies is | Very unimportant -3 -2 -1 0 1 2 3 Very important |
| The agricultural technicians think that control of rabies is | Very unimportant -3 -2 -1 0 1 2 3 Very important |
| **Motivation to comply** |  |
| Does the opinion of the following people regarding reduction of rabies influence your intention to implement preventive measures? |  |
| Veterinarians | Very unimportant -3 -2 -1 0 1 2 3 Very important |
| The health extension technician | Very unimportant -3 -2 -1 0 1 2 3 Very important |
| My family | Not at all 1 2 3 4 5 6 7 Very much |
| The agricultural technicians | Not at all 1 2 3 4 5 6 7 Very much |

| **Perceived Behavioural Control for vaccinating dog fair price** |  |
| --- | --- |
| **Strength of Control Beliefs** |  |
| Vaccination of dog is difficult | Strongly disagree 1 2 3 4 5 6 7 Strongly agree |
| It takes time for vaccination of dogs | Strongly disagree 1 2 3 4 5 6 7 Strongly agree |
| Vaccination of dog is expensive | Strongly disagree 1 2 3 4 5 6 7 Strongly agree |
| **Power of Factors to Influence the Behaviour** |  |
| I know how to vaccinate a dog | Strongly disagree 1 2 3 4 5 6 7 Strongly agree |
| I have time to vaccinate a dog | Strongly disagree 1 2 3 4 5 6 7 Strongly agree |
| I can cover the cost for vaccinating a dog | Strongly disagree 1 2 3 4 5 6 7 Strongly agree |

**Intention: Leashing of dogs**

| In the near future I plan to leash my dog to reduce rabies in my village | Strongly disagree -3 -2 -1 0 1 2 3 Strongly agree |
| --- | --- |

| **Attitude for Leashing of dogs** |  |
| --- | --- |
| **Behavioural beliefs** |  |
| Tying of a dog can prevent a dog from becoming infected with rabies | Strongly disagree 1 2 3 4 5 6 7 Strongly agree |
| Tying a dog prevent the transmission of rabies from one dog to another | Strongly disagree 1 2 3 4 5 6 7 Strongly agree |
| Tying a dog prevent rabies transmission from dog to person | Strongly disagree 1 2 3 4 5 6 7 Strongly agree |
| **Outcome evaluation** |  |
| Restricting of dog movement by tying is so useful to prevent a dog from becoming infected with rabies | Strongly disagree -3 -2 -1 0 1 2 3 Strongly agree |
| Tying a dog useful to reduce the transmission of rabies from one dog to another | Strongly disagree -3 -2 -1 0 1 2 3 Strongly agree |
| Tying a dog is good way to prevent the transmission of rabies | Strongly disagree -3 -2 -1 0 1 2 3 Strongly agree |

| **Subjective Norm for Leashing of dogs** |  |
| --- | --- |
| What, according to your knowledge, is the opinion of the following people regarding the control of rabies |  |
| Veterinarians think that leashing of dog is | Very unimportant 1 2 3 4 5 6 7 Very important |
| The health extension technician thinks that leashing of dog is | Very unimportant 1 2 3 4 5 6 7 Very important |
| The agriculture technician think that leashing of dog is | Very unimportant 1 2 3 4 5 6 7 Very important |
| My family think that leashing of dog is | Very unimportant 1 2 3 4 5 6 7 Very important |
| **Motivation to comply** |  |
| Does the opinion of the following people regarding leashing of dogs influence your intention to leash dogs? |  |
| Veterinarians | Not at all -3 -2 -1 0 1 2 3 Very much |
| The health extension technicians | Not at all -3 -2 -1 0 1 2 3 Very much |
| The agriculture sectore/technician | Not at all -3 -2 -1 0 1 2 3 Very much |
| My family | Not at all -3 -2 -1 0 1 2 3 Very much |
| **Perceived Behavioural Control for leashing dog** |  |
| **Strength of Control Beliefs** |  |
| Tying a dog is difficult | Strongly disagree 1 2 3 4 5 6 7 Strongly agree |
| Tying a dog is take time | Strongly disagree 1 2 3 4 5 6 7 Strongly agree |
| Material for tying a dog is expensive | Strongly disagree 1 2 3 4 5 6 7 Strongly agree |
| **Power of Factors to Influence the Behaviour** |  |
| I know how to tie a dog | Strongly disagree -3 -2 -1 0 1 2 3 Strongly agree |
| I have time to tie my dog | Strongly disagree -3 -2 -1 0 1 2 3 Strongly agree |
| I can afford to cover the cost of buying a dog harness? | Strongly disagree -3 -2 -1 0 1 2 3 Strongly agree |
