# Supplementary material for: Dog owners’ intention to control rabies and their willingness to pay for rabies vaccine in Northwestern Ethiopia
Source: PLOS Glob Public Health. 2025 Mar 11;5(3):e0003974. doi: 10.1371/journal.pgph.0003974 (PMC11896628; doi:10.1371/journal.pgph.0003974)
Supplement: S1 Appendix — (DOCX) [file pgph.0003974.s002.docx]

## **Questionnaire for willingness to pay survey**

Date ፡_____/_____/_____

Dear respondent,

The purpose of this questionnaire is to obtain data for the research “Dog owners’ intention to control rabies and their willingness to pay for rabies vaccine in northwestern Ethiopia” using Contingent evaluation method. The aims of the study were to assess dog owners WTP for rabies vaccine, and to identify factors influencing their WTP.

Your response is important for the success of the study. So, I kindly request your willingness to answer questions I am going to ask you. Your response will be confidential; I assure you that the data will be analyzed and reported anonymously.

Please be aware that you have the right to stop or withdraw from the study with any reason.

Thank you in advance for your willingness to participate in the study.

Name_______________________Woreda፡ _____________kebele_____________

Sex: _____ Age______Level of education_____Family_________

Job፡ _______________________________________

Information gathering about rabies and vaccination awareness

1/ Do you know the presence rabies?

A/ No B/ Yes C/ I don’t know

1.1/ If yes from whom did you get information about rabies____________________________

2. What are the symptoms of rabies?_________________________A/Fear of water B/changing behavior C/restlessness D/salivation E/paralysis F/aggressiveness and others_____________

3/ what do you do if your dog gets rabies?________________________________

4. Did you know that rabies can be transmitted from dog to dog and from dog to human?

A/ No B/ Yes. If yes, how it can transmitted____________________________

5/ what happens to dogs with rabies?

A/ Dead B/ Recovered by medication and treatment C/ I don’t know

The most important way to prevent rabies is to vaccinate dogs before they develop rabies. Since the rabies vaccine is purchased, it can be provided by the government; otherwise it can be purchased with private funds. The first rabies vaccine is given when the dog is above 3 months old, and the second round of vaccination is repeated when the dog is one to one and a half years old, after which it should be vaccinated once every 3 years. A dog vaccinated this way is 99%-100% rabies free. Therefore, rabies can be prevented by vaccinating dogs

Did you fully understand what was explained above about the vaccine? (If you don't understand, repeat the above. If you understand, continue the question from one group.

| Group 1 Respondents | 1. Are you willing to pay 20 Birr to vaccinate your dog against rabies?  A/ Yes B/No  If yes, are you willing to pay if the cost of the vaccine (increase by 25% from above) is 25 ETB  A/ Yes B/No  If no, are you willing to pay if the price of the vaccine (25% lower than the above) is 15 Birr?  / Yes B/No |
| --- | --- |
| Group 2 Respondents | 1. Are you willing to pay 30 Birr to vaccinate your dog against rabies?  A/ Yes B/No  If yes, are you willing to pay if the cost of the vaccine (increase by 25% from above) is 37.50 ETB  A/ Yes B/No  If no, are you willing to pay if the price of the vaccine (25% lower than the above) is 22.50 Birr?  / Yes B/No |
| Group 3 Respondents | 1. Are you willing to pay 40 Birr to vaccinate your dog against rabies?  A/ Yes B/No  If yes, are you willing to pay if the cost of the vaccine (increase by 25% from above) is 50 ETB  A/ Yes B/No  If no, are you willing to pay if the price of the vaccine (25% lower than the above) is 30 Birr?  / Yes B/No |
| Group 4 Respondents | 1. Are you willing to pay 50 Birr to vaccinate your dog against rabies?  A/ Yes B/No  If yes, are you willing to pay if the cost of the vaccine (increase by 25% from above) is 62.50 ETB  A/ Yes B/No  If no, are you willing to pay if the price of the vaccine (25% lower than the above) is 37.50 Birr?  / Yes B/No |

**Domestic dog information**

Sex of dogs: __________Number of dogs________Age_________

2 Has your dog been vaccinated against rabies in the last 2 years?

A/ Yes B/ No

3/What kind of care do you take for your dog(s)?

What kind of care does the community in this area provide for the dog(s)?

_______________________________________________________________

_______________________________________________________________
